# Molecular Screening of *Plasmodium* spp. in Free-Living Ring-Tailed Coatis (*Nasua nasua*) and Nine-Banded Armadillos (*Dasypus novemcinctus*) in the Peruvian Amazon

**DOI:** 10.3390/ani15162413

**Published:** 2025-08-18

**Authors:** Gabriela M. Ulloa, Alex D. Greenwood, Omar E. Cornejo, Frederico Ozanan Barros Monteiro, Meddly L. Santolalla, Pedro Mayor

**Affiliations:** 1 Departament de Sanitat i d’Anatomia Animals, Facultat de Veterinària, Universitat Autònoma de Barcelona (UAB), 08193 Barcelona, Spain; 2 Programa de Pós-Graduação em Saúde e Produção Animal na Amazônia, Universidade Federal Rural da Amazônia (UFRA), Av. Presidente Tancredo Neves 2501, Terra Firme, Belém 66077-830, Pará, Brazil; frederico.monteiro@ufra.edu.br; 3 Grupo de Enfermedades Infecciosas Re-Emergentes, Universidad Científica del Sur, Villa el Salvador, Lima 15067, Peru; 4 Leibniz-Institute for Zoo and Wildlife Research, Alfred-Kowalke-Strasse 17, 10315 Berlin, Germany; greenwood@izw-berlin.de; 5 Department of Veterinary Medicine, Freie Universität Berlin, Oertzenweg 19b, 14163 Berlin, Germany; 6 Department of Ecology and Evolutionary Biology, University of California Santa Cruz, Santa Cruz, CA 95064, USA; omcornej@ucsc.edu; 7 Emerge, Research Unit on Emerging Diseases and Climate Change, Universidad Peruana Cayetano Heredia, Lima 15015, Peru; meddly.santolalla.r@upch.pe; 8 Comunidad de Manejo de Fauna Silvestre en la Amazonía y en Latinoamérica (COMFAUNA), 332 Malecon Tarapaca, Iquitos 16006, Peru; 9 Museo de Culturas Indígenas Amazónicas, Loreto, Iquitos 16006, Peru

**Keywords:** wildlife, malaria, zoonotic parasites, wildlife hosts, *Plasmodium*, One Health

## Abstract

Identifying the diversity of wildlife hosts for malaria parasites in wildlife is crucial for understanding transmission dynamics in endemic regions where humans, vectors, and wildlife heavily overlap. We examined the presence of *Plasmodium* parasites in free-ranging ring-tailed coatis (*Nasua nasua*, *n* = 44) and nine-banded armadillos (*Dasypus novemcinctus*, *n* = 66) from an Indigenous community in the Peruvian Amazon. Nested PCR targeting the mitochondrial *cytb* gene detected *Plasmodium* spp. DNA in two coatis (4.7%). Sequencing revealed one lineage identical to *Plasmodium vivax/P. simium* and another to *P. malariae/P. brasilianum*. A subset of samples was reanalyzed using *cox3*-based PCR and sequencing in an independent laboratory, confirming *P. malariae/P. brasilianum* in one coati. No infections were observed in armadillos. These results indicate that coatis in the wild may host diverse Plasmodiidae parasites and that coatis may even carry *Plasmodium* spp., likely as incidental hosts. Expanding surveillance to additional non-primate mammals will help clarify their role in sylvatic malaria ecology and evaluate potential zoonotic risks.

## Simple Summary

Malaria is a disease caused by *Plasmodium* parasites, which are usually transmitted between mosquitoes and humans or non-human primates (NHPs). However, the role of other wild mammals as carriers remains poorly understood, especially in remote tropical forests. We analyzed blood samples from free-ranging ring-tailed coatis (*Nasua nasua*) and nine-banded armadillos (*Dasypus novemcinctus*) hunted for subsistence in an Indigenous community of the Peruvian Amazon. DNA analysis revealed that coatis carried lineages identical to *Plasmodium vivax/P. simium*, the most common cause of human malaria in the region, and *P. malariae/P. brasilianum*, a species frequently found in Neotropical NHPs. These findings suggest that wild coatis may occasionally become infected with malaria parasites closely related to those infecting humans and NHPs. This study highlights the importance of extending wildlife surveillance beyond primates to improve understanding of malaria transmission at the human–wildlife interface.

## 1. Introduction

Malaria constitutes a significant concern for public and global health, particularly in forested regions where close interactions among humans, mosquito vectors, and wildlife influence disease dynamics [1,2,3]. The disease results from protozoan parasites of the genus *Plasmodium* (Apicomplexa: Plasmodiidae), which are vector-borne and exhibit extensive ecological and host diversity [4]. Although *Plasmodium* spp. are extensively studied in humans and non-human primates, the genus encompasses numerous lineages that infect other vertebrates—including birds and reptiles—with varying degrees of pathogenicity and host specificity [5].

However, the diversity of this parasite genus is noteworthy, with primary focus directed toward humans and non-human primates [6,7,8]. Although malaria remains an extensively researched pathogen, there exists a gap in understanding regarding the ecological role of non-primate mammals within its lifecycle. Consequently, elucidating parasite–host relationships is essential for clarifying the disease dynamics of malaria and the evolutionary interactions within their hosts and ecosystems. This issue is particularly pertinent in endemic and neglected regions along national borders, characterized by nomadic human populations, where interactions between wildlife and vectors are frequent, and such areas serve as hotspots for host–pathogen–vector interactions [9,10].

Host specificity is a key factor influencing parasite virulence [11,12]. In highly diverse environments such as the Amazon, elevated biodiversity reduces the risk of transmission by dispersing bites among multiple hosts, many of which are non-competent, thereby exhibiting a dilution effect [13]. Host–parasite relationships are not solely dictated by physiological compatibility and immune constraints but are also shaped by ecological factors such as vector distribution, host abundance, and transmission opportunities between species [14,15]. While specialization may facilitate reproductive success and genetic diversification within a limited host range, generalist strategies can enhance resilience to fluctuations in host populations and enable a broader geographical range [16]. These evolutionary trade-offs contribute to the variability observed even among phylogenetically closely related *Plasmodium* lineages [17,18].

Molecular studies of avian hemosporidia have yielded important insights into the diversity and host range plasticity of *Plasmodium*. Mitochondrial markers have identified intraspecific lineages within natural environments characterized by coinfections and high species richness [19,20,21]. These findings emphasize that local wildlife, including terrestrial and hunted birds, harbor a remarkable and inadequately explored diversity of blood parasites, including lineages that likely represent distinct biological species [22]. This complexity is presumably reflected in non-primate mammalian hosts, although this area has been insufficiently studied.

Among potential mammalian hosts in the Neotropics, the South American ring-tailed coati (*Nasua nasua*) and the nine-banded armadillo (*Dasypus novemcinctus*) are of particular interest. Both species are abundant, widely distributed, and frequently hunted for subsistence, making them ideal candidates for investigating the wildlife cycle of vector-borne pathogens [23,24]. *Nasua nasua* is a diurnal, omnivorous procyonid distributed in the tropical and subtropical forests of South America, including tropical rainforests, dry scrublands, savannas, and Chaco habitats [25,26]. It generally avoids flooded areas and may play an essential ecological role in mainland forests due to its gregarious behavior and feeding patterns [27,28]. The IUCN classifies this species as ‘Least Risk and Least Concern’ [29] due to habitat loss due to deforestation and hunting.

In contrast, *D. novemcinctus* is a nocturnal, solitary xenarthran with a wide distribution ranging from the southern United States to northern Argentina [30]. Although classified as ‘Least Concern’ by the IUCN [31], it is subject to intensive hunting in Amazonian and rural regions for its meat and use in traditional medicine [32]. The reproductive biology of the armadillo, polyembryony, which results in genetically identical offspring, and its known susceptibility to multiple human pathogens, including *Mycobacterium leprae*, make it a well-established model organism in biomedical research [30,33]. It is important to note that both coatis and armadillos have previously been implicated as possible reservoirs or incidental hosts of other vector-borne pathogens, including *Trypanosoma cruzi*, *Leishmania* spp., and filarial nematodes [34,35].

Our objective was to investigate the presence of *Plasmodium* spp. in *N. nasua* and *D. novemcinctus* from a remote Indigenous territory in the northeastern Peruvian Amazon, where both species are frequently hunted by community members for subsistence [23]. We used molecular tools targeting the mitochondrial genes *cytb* and *cox3* to detect and identify parasite lineages from blood samples. This study contributes to the growing effort to understand the broader ecological spectrum of wild malaria, especially in neotropical areas with high biodiversity but little monitoring. Our findings expand the range of vertebrates known to be related to *Plasmodium* spp. and add to the basic knowledge needed to clarify the evolutionary origins and transmission potential of blood parasites in non-primate mammals.

## 2. Materials and Methods

### 2.1. Study Area

This study was conducted in an area covering 107,000 hectares of predominantly upland forest located along the Yavari-Mirin River basin in the northeastern Peruvian Amazon. The Yavari-Mirin River basin, which supplies the middle section of the Yavari River, constitutes a highly diverse landscape, encompassing upland forests with nutrient-poor sandy soils as well as flooded forests with comparatively nutrient-rich soils [23]. The Yavari-Mirin River basin is located 302 km from Iquitos, the nearest city, and harbors a high wildlife diversity, particularly of mammals.

The site is exclusively inhabited by the Indigenous Yagua community of Nueva Esperanza (04°19′53″ S, 71°57′33″ W; UT-5:00), with 329 inhabitants (159 men and 170 women) in 2019 [36]. Villagers rely on subsistence activities such as hunting, fishing, forestry, and small-scale agriculture, supplementing their livelihoods with occasional trade in timber, fish, wild meat, and agricultural products [24]. Accessibility to urban areas is difficult but has increased with more frequent logging traffic, facilitating the introduction of urban customs that require monetary income, such as electrical appliances. Thus, activities with an economic return, such as timber extraction, are attractive. Although it is uncommon for residents to travel outside of the village, when they choose to travel, it is usually for short trips to nearby locations, which are primarily motivated by trade-related activities. The Peruvian Ministry of Health designates this study area as a low but stable malaria-endemic area [37].

The climate of the region is typically equatorial, with annual temperatures ranging from 22 to 36 °C, relative humidity levels of 80–100%, and annual rainfall of 1500–3000 mm [23].

### 2.2. Samples

From 2007 to 2020, local hunters collected blood samples from the cranial or caudal vena cava from 43 ring-tailed coatis and 66 nine-banded armadillos and impregnated FTA^®^ cards (Scheilcher & Schuell, Dassel, Germany) with the blood. Local hunters were also trained to record the date, species, and sex of all animals of all taxa hunted as part of their normal subsistence activities. There was no incentive, financial or otherwise, to encourage additional hunting of wildlife. This sampling strategy was integrated into a community participatory program to improve the conservation and sustainability of natural resources and indigenous livelihoods [24]. This approach allowed for the determination of wildlife population health status using discarded material from legal subsistence hunting.

Dry blood spots were sealed in individual plastic bags with desiccant and stored in the collection area at room temperature for a minimum of two weeks and a maximum of six months before being transferred to a −70 °C freezer for preservation. DNA was extracted from blood spots on filter paper using the AllPrep DNA/RNA Mini Kit (Qiagen, Hilden, Germany) according to the manufacturer’s instructions at the Malaria Laboratory of the Institute of Global Health, Barcelona.

### 2.3. Ethical Review

The research protocols for the sampling of wild animals were approved by the Peruvian Forest and Wildlife Service (No. 258-2019-MINAGRI-SERFOR-DGGSPFFS) and the Institutional Animal Use Ethics Committee of the Universidad Peruana Cayetano Heredia (No. 102142). Dried wildlife blood samples on filter paper were exported from Peru to Spain with the approval of the Ministry of Agriculture and Irrigation (MINAGRI) through the Peruvian Forestry and Wildlife Service—SERFOR (No. 003258/SP, No. 003260/SP, No. 003568-SERFOR, No. 003579-SERFOR) according to the Nagoya Protocol.

### 2.4. Amplification of Plasmodium Sequences by Polymerase Chain Reaction (PCR)

Detection of *Plasmodium* DNA was performed using a nested PCR (nPCR) protocol targeting the *cytb* gene, previously applied in wildlife hosts [36], with modifications implemented to enhance sensitivity in samples potentially exhibiting low parasitemia [38,39].

The *cytb* nPCR facilitates the specific detection of members of the family Plasmodiidae and enables discrimination at the genus level. The initial PCR cycle amplified a fragment of approximately 776 base pairs utilizing primers DW2 (5′-TAA TGC CTA GAC GTA TTC CTG ATT ATC CAG-3′) and DW4 (5′-TGT TTG CTT GGG AGC TGT AAT CAT AAT GTG-3′), while the subsequent round employed *Plasmodium*-specific primers FP3 (5′-TAT ATA ACT TAT TTT TTG ATA TG-3′) and RP3 (5′-GTR ATW GCA TTA TCT GGA TGT GA-3′).

For the initial round, reactions (20 μL final volume) comprised 5 μL of template DNA, 10 μL of 2× Platinum™ II Hot-Start PCR Master Mix (Invitrogen, Carlsbad, CA, USA), and 0.5 μM of each primer. Thermal cycling included an initial denaturation at 94 °C for 3 min, followed by 40 cycles of denaturation at 94 °C for 20 s, annealing at 62 °C for 30 s, and extension at 72 °C for 1 min, concluding with a final elongation step at 72 °C for 10 min.

The second PCR cycle utilized 2 μL of a 1:40 dilution of the initial-round product as the template, with the reaction composition remaining unchanged except for an annealing temperature of 54 °C. The cycling conditions were otherwise identical.

Positive controls consisted of DNA derived from the *P. falciparum* 3D7 strain, whereas negative controls contained molecular-grade water. Amplified products were purified using the QIAquick Gel Extraction Kit (Qiagen, Germany) and sequenced by Macrogen (Seoul, Republic of Korea) with FP3 and RP3 primers to confirm *Plasmodium* identity.

### 2.5. Validation of Plasmodium *spp.* Detection

To independently confirm *Plasmodium* infections detected by *cytb*-based nPCR, we re-tested a subset of eight dried blood spot samples at the Leibniz Institute for Zoo and Wildlife Research (IZW, Berlin, Germany). This subset included four previously positive samples (two coatis and two armadillos) and four randomly selected negatives.

Validation was performed using a nested PCR targeting the *cox3* gene, following a protocol previously applied with modifications to improve species-level discrimination in wildlife malaria specificity [40]. The *cox3* assay amplifies the genus *Plasmodium* in the first round, followed by species-specific reactions in the second round for *P. falciparum* (Pf), *P. vivax/P. simium* (Pv/Ps), and *P. malariae/P. brasilianum* (Pm/Pb). Primer sequences were selected to include ≥7 mismatches at the 3′ end relative to non-target species, enhancing specificity.

First-round reactions (25 µL) contained 5 µL of template DNA, 2 µM of each genus-specific primer, and 12.5 µL of 2X MyTaq Master Mix (Meridian Bioscience, Newtown, OH, USA). Cycling conditions were as follows: initial denaturation at 96 °C for 1 min; 40 cycles of 96 °C for 10 s, 63 °C for 1 min, and 72 °C for 1 min; final extension at 72 °C for 10 min.

Nested reactions were run separately for each target species group using 2.5 µL of first-round product diluted 1:50, with 4 µM of each primer and 10 µL of 2X MyTaq Mix. Pf and Pv/Ps assays used annealing at 54 °C for 1.5 min, whereas Pm/Pb used 58 °C. All nested PCRs included molecular-grade water as negative controls.

Amplicons were gel-purified and Sanger-sequenced. Sequences were aligned to GenBank references to confirm species identity. This validation method was chosen because *cox3* provides greater phylogenetic resolution than *cytb* for specific *Plasmodium* species and allows clear differentiation between species related to human malaria in South America.

### 2.6. Phylogenetic Analysis

Mitochondrial DNA alignments were generated using the CLUSTAL-OMEGA algorithm [41] as implemented in Geneious Prime (v. 2025.1.3, Biomatters Ltd., Auckland, New Zealand) [42]. Two alignments were constructed: one for partial *cytb* and another for partial *cox3* sequences, each including 19 reference sequences of *Plasmodium* species found in humans, non-human primates, rodents, and birds, along with one sequence obtained from the coati sample in this study. Bayesian inference (BI) was performed using MrBayes 3.2.6 [43], also implemented in Geneious Prime.

The substitution model GTR + Γ + I (general time-reversible model with gamma-distributed rate heterogeneity and a proportion of invariant sites) was selected based on the lowest Bayesian Information Criterion (BIC) values estimated in MEGA v. 11.0.13 [44]. Analyses were run with two simultaneous chains for one million generations, sampling every 500 generations and discarding the first 25% of trees as burn-in. Convergence was confirmed by examining the adequate sample size (ESS) and posterior distributions. Posterior probabilities were calculated for each node. *Leucocytozoon dubreuili* (KY653795.1) was used as the outgroup in both phylogenies.

### 2.7. Statistical Analysis

All analyses were conducted using R version 4.4.2 (R Core Team, Vienna, Austria, 2024). The proportion of samples that tested positive for *Plasmodium* was determined for each host species and molecular assay. The concordance between *cytb* and *cox3* PCR results within the subset of coati samples tested with both markers was assessed by calculating the sensitivity, specificity, positive predictive value (PPV), negative predictive value (NPV), and Cohen’s Kappa coefficient, in addition to the corresponding percentage agreement.

### 2.8. Data Availability

The data supporting the findings of this study are available in the CORA Repositori de Dades de Recerca Support (https://doi.org/10.34810/data2474, accessed on 13 August 2025). The partial *cytb* sequences corresponding to *P. malariae/P. brasilianum* (sample coati5013) and *P. vivax/P. simium* (sample coati5054) were deposited in GenBank under accession numbers PV868253 and PV868254, respectively. Additionally, the partial *cox3* sequence confirming the presence of *P. malariae/P. brasilianum* in the same coati (sample coati5013), obtained through an independent validation assay, was deposited under accession number PV862362.

## 3. Results

Blood samples from 43 ring-tailed coatis (*Nasua nasua*) and 66 nine-banded armadillos (*Dasypus novemcinctus*) were analyzed via nested PCR to detect the presence of Plasmodiidae DNA. In the initial round of the *cytb* assay, amplification was observed in two out of 43 coatis (4.7%) and two out of 66 armadillos (3.0%). However, only the two coati samples yielded amplification during the subsequent nested PCR targeting *Plasmodium* spp., thereby confirming genus-level detection in these individuals. None of the armadillo samples tested positive in the genus-specific nested step, and further sequencing was not possible for this species.

Sequencing of the *cytb* amplicons revealed that the two positive coatis exhibited 100% nucleotide identity with reference sequences of *P. vivax/P. simium* (GenBank accessions LT635627.1, NC_007243.1, AY791517.1, and AY722798.1) for sample coati5054 and *P. malariae/P. brasilianum* (GenBank accessions AB354570.1 and GQ355484) for sample coati5013. The amplified fragment sizes were 790 bp (coati5013) and 837 bp (coati5054), and chromatograms demonstrated no evidence of double peaks or mixed infections. These findings suggest the presence of two distinct *Plasmodium* lineages in the coatis examined.

**Figure 1:**
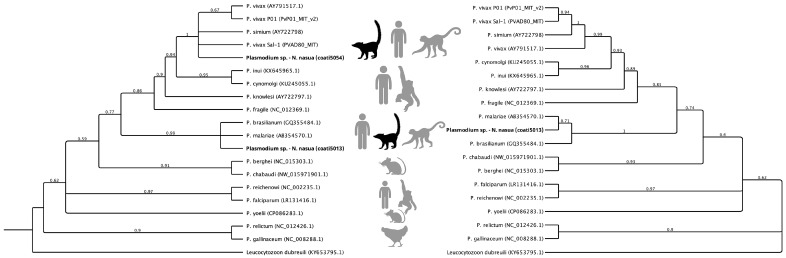
Bayesian phylogenetic trees based on partial mitochondrial *cytb* (**left**) and *cox3* (**right**) gene sequences from *Plasmodium* spp., including lineages detected in two free-ranging coatis (*Nasua nasua*) from the Yavarí-Mirin River basin in the northeastern Peruvian Amazon. The coati sequences are highlighted in bold. Trees were inferred using MrBayes with one million generations, and posterior probabilities are shown at each node. *Leucocytozoon dubreuili* was used as an outgroup. The trees illustrate the genetic relationships of the coati-derived sequences to human and non-human primate *Plasmodium* species.

To validate these findings, a subset of eight samples was reanalyzed using nested *cox3* PCR in an independent laboratory, including two *cytb*-positive coatis, two *cytb*-negative coatis from the same locality, and four negative controls. The *cox3* amplicon was sequenced and compared to GenBank references (accession numbers: AB354570.1 and GQ355484). Only the sample coati5013 (234 bp) was confirmed as *P. malariae/P. brasilianum*, showing 100% identity with the same references as in the *cytb* analysis. This sample was the only one included in the phylogenetic reconstruction (Figure 1). The *P. vivax/P. simium*-positive sample (coati5054) from the *cytb* assay did not amplify with *cox3* primers. All *cytb*-negative samples remained negative in the *cox3* assay (Table 1).

The phylogenetic trees (Figure 1) illustrate the placement of the coati-derived sequences among *Plasmodium* lineages infecting humans, non-human primates, rodents, and birds. In the *cytb* phylogeny (Figure 1, left), the coati sequence coati5054 clustered within the *P. vivax/P. simium* clade (posterior probability = 0.98), grouping closely with human and non-human primate isolates from South America. The second coati sequence (coati5013) grouped with the *P. malariae/P. brasilianum* clade (posterior probability = 1.00), alongside both human and primate reference sequences from the Neotropics. In the *cox3* phylogeny (Figure 1, right), only the coati5013 sequence was recovered, maintaining its position within the *P. malariae/P. brasilianum* cluster (posterior probability = 1.00) and confirming the congruence between mitochondrial markers. Branch lengths in both trees indicate a close genetic relationship between the coati-derived sequences and their closest reference strains, in agreement with the 100% nucleotide identity observed. The consistent topology across markers reinforces the reliability of lineage identification and supports the interpretation that these infections correspond to well-characterized *P. vivax/P. simium* and *P. malariae/P. brasilianum* lineages circulating in South America.

To assess the agreement between the two mitochondrial markers, a Kappa analysis was performed on the eight coati samples tested with both *cytb* and *cox3*. The agreement was moderate (Kappa = 0.48), with a sensitivity of 0.50, a specificity of 1.00, a PPV of 1.00, and an NPV of 85.7%. Although the number of positive samples was low, this analysis provided a descriptive measure of concordance between the two assays in this dataset.

## 4. Discussion

Research concerning *Plasmodium* in non-human mammals has yielded critical insights into the adaptability and evolutionary trajectories of these parasites across diverse vertebrate hosts [45,46]. In this study, we employed nested PCR techniques to identify genetic markers of the Plasmodiidae family in blood samples obtained from ring-tailed coatis and nine-banded armadillos within their natural habitat in the Peruvian Amazon. To our knowledge, this represents the first detection of Plasmodiidae DNA in these coatis, suggesting their potential, albeit incidental, participation in the intricate and heterogeneous malaria transmission dynamics characteristic of the region [47,48,49].

We identified a *cytb* sequence in one coati that was 100% identical to *P. vivax*/*P. simium*, and a second coati tested positive for *P. malariae/P. brasilianum* in both *cytb*- and *cox3*-based PCRs performed in different laboratories. These molecular findings suggest that ring-tailed coatis may occasionally harbor *Plasmodium* lineages typically associated with humans or Neotropical primates [50,51,52,53]. It is important to clarify that the nested *cytb* PCR used in this study is a genus-specific assay targeting the mitochondrial DNA of *Plasmodium* spp. This method has been applied to various mammalian hosts, including ungulates, and is not limited to *Plasmodium* species infectious to primates [36,54,55]. Therefore, although our sequences aligned with known primate parasites, the assay itself has the potential to detect other lineages of *Plasmodium*, including those adapted to non-primate hosts.

Although only one of the *Plasmodium*-positive samples was confirmed by both genetic markers, the concordant results are in accordance with infection of the individual coati. The other sample that tested positive for *cytb* was not successfully amplified using *cox3*, which likely reflects variations in assay sensitivity, a low parasite load, or discrepancies in the binding regions of the *cox3* primer, rather than the actual absence of the lineage in that individual [56]. The nested PCR for *cox3* employed for validation is also specific to the genus *Plasmodium* but has been primarily optimized for human malaria lineages [40,57]. Its objective was to verify the positive results obtained for *cytb* independently and to confirm the species identity where feasible, rather than to exclude other *Plasmodium* species.

The selection of mitochondrial markers (*cytb* and *cox3*) over the more commonly employed *18S ribosomal RNA* gene was a deliberate decision. Although *18S* is highly conserved, exists in multiple copies, and is frequently utilized for the identification of *Plasmodium* species associated with human malaria, its conserved nature may restrict the phylogenetic resolution among closely related species and lineages, particularly in wild hosts where novel or divergent strains could be present [56,57]. Additionally, the mitochondrial genome of *Plasmodium* exhibits a substantially higher copy number per parasite (20–150 copies) in comparison to *18S ribosomal RNA* (4–8 copies) [40]. This increased template abundance potentially enhances amplification efficiency, diminishes the necessity for multiple nested amplification rounds, and augments detection sensitivity, especially in samples exhibiting low parasitemia [36]. Mitochondrial genes evolve at a faster rate, affording greater resolution for species-level discrimination and phylogenetic analysis [58]. Moreover, *cytb* and *cox3* are extensively represented within public databases for both human and non-human *Plasmodium* species, facilitating direct comparisons with well-characterized lineages [59]. Therefore, the mitochondrial markers used in this study offered an ideal balance between broad genus-level detection and improved sensitivity due to the higher template copy number.

Zoonotic exchanges involving *Plasmodium* have been documented in several South American settings, particularly between humans and non-human primates [53,60]. Moreover, *P. falciparum* DNA has been detected in free-ranging rodents (*Oligoryzomys* sp.), and sequences similar to *P. malariae* have been reported in the red brocket deer (*Mazama americana*), suggesting that a broader spectrum of mammalian taxa may sporadically harbor human malaria parasites [36,61]. Although the detection of *Plasmodium* DNA alone does not confirm active infection or parasite replication, it indicates parasite presence and raises the possibility that interactions among the host, vector, and parasite may have been underestimated in forest environments [11,52].

An additional consideration is that members of the Plasmodiidae family include genera beyond *Plasmodium* sensu stricto, such as *Polychromophilus*, *Haemoproteus*, and *Leucocytozoon* [62]. Although the *cytb* and *cox3* regions utilized here were designed for *Plasmodium*, the amplification of other members within the Plasmodiidae family remains theoretically feasible, particularly if novel or divergent mitochondrial sequences are present within the examined host species [63]. Our sequences aligned unambiguously with *Plasmodium* spp. in GenBank; however, in wildlife surveillance, especially concerning poorly studied taxa such as armadillos, the potential for detecting other Plasmodiidae lineages should be acknowledged. Future research employing fewer taxon-specific primer sets and sequencing methodologies, such as metabarcoding or whole-genome sequencing, may elucidate whether non-*Plasmodium* genera are circulating within these hosts.

The detection of Plasmodiidae DNA in coatis and armadillos expands our understanding of the possible diversity of hosts involved in wild human malaria systems. Both species have previously been recognized as hosts of various other apicomplexan parasites [27,28,34,35], which lends support to their susceptibility to hemoparasites and justifies their inclusion in comprehensive surveillance initiatives. Concerning armadillos, although infections with the second genetic marker were not confirmed, the initial positive finding for *cytb* merits further investigation. Considering their fossorial lifestyle and potential exposure to different mosquito species in comparison to arboreal or terrestrial primates, armadillos may harbor distinct *Plasmodium* lineages occupying ecological niches that are as yet uncharacterized.

The findings of the study highlight the significance of broadening malaria surveillance efforts beyond primates to encompass other wildlife species that may serve an incidental or secondary function in sustaining the parasite. The application of integrative molecular methodologies, including multilocus detection and phylogenetic tools, is crucial for a comprehensive understanding of host–parasite relationships and transmission dynamics within the wildlife cycle [4,62,64]. Nevertheless, research concerning zoonotic malaria has primarily concentrated on the human–primate interface, thereby leaving a notable gap in our understanding of the potential roles of other mammals in the parasite’s ecology. Incorporating non-primate mammals into surveillance strategies could uncover concealed transmission routes, identify novel parasite diversity, and enhance predictive models for disease emergence [1,65].

The broader role of wildlife as a reservoir or as incidental hosts for zoonotic pathogens, including parasites, bacteria, and viruses, is increasingly being recognized [33,66]. Continuous molecular surveillance of wildlife populations, using multiple genetic markers, will be essential to test specific hypotheses about the role of different species in the ecology of *Plasmodium*. These efforts are particularly relevant in tropical ecosystems, where the overlap between humans, vectors, and wildlife creates opportunities for silent transmission cycles and the possible emergence of novel parasites [67].

In the Peruvian Amazon, where subsistence hunting of coatis and armadillos is prevalent, understanding the parasite communities associated with these species is also pertinent from a food security standpoint [68]. Although *Plasmodium* spp. are not recognized as being transmitted via consumption, the occurrence of hemoparasites in wild animal meat underscores the necessity for integrated One Health approaches that consider both vector-borne and alternative transmission risks [69].

Finally, our findings underscore both the potential and limitations of existing molecular surveillance tools for malaria in wildlife. While mitochondrial markers have provided significant resolution and confirmed the genus identity of the detected lineages, the absence of amplification in specific samples emphasizes the necessity for supplementary assays to encompass the full spectrum of parasitic infections. Future research should also focus on the vector component, identifying the mosquito species that feed on coatis and armadillos and assessing their competence in transmitting the detected lineages. Such efforts will address critical gaps in understanding *Plasmodium* ecology and its implications for wild malaria epidemiology.

## 5. Conclusions

Although active transmission could not be definitively confirmed, the detection of *Plasmodium* DNA in coatis suggests their potential function as incidental hosts within sylvatic malaria cycles. These findings highlight the importance of including non-primate mammals in surveillance initiatives, thereby expanding the range of host diversity considered in malaria ecology. Augmenting such efforts is essential for assessing zoonotic risks and formulating more comprehensive control strategies, especially in regions where close interactions among humans, vectors, and wildlife may perpetuate previously unrecognized transmission pathways.

## Figures and Tables

**Table 1:** Summary of molecular results for *Plasmodium* spp. detection in free-ranging coatis and armadillos from the Yavarí-Mirin River basin, northeastern Peruvian Amazon.

Host	N	*cytb* nPCR Positive (Round 1)	*cytb* nPCR *Plasmodium* (Round 2)	*cox3* nPCR Validated	Confirmed Species
*Nasua nasua*	43	2 (4.7%)	2 (4.7%)	1/4	*P. malariae-like*
*Dasypus novemcinctus*	66	2 (3.0%)	0	0/4	None

## Data Availability

The data used to support the findings of this study are available at the CORA Repositori de Dades de Recerca Support (https://doi.org/10.34810/data2474).
